# The subnivium, a haven for *Trichinella* larvae in host carcasses

**DOI:** 10.1016/j.ijppaw.2019.02.007

**Published:** 2019-03-04

**Authors:** Luca Rossi, Maria Interisano, Gunita Deksne, Edoardo Pozio

**Affiliations:** a Department of Veterinary Sciences, University of Turin, Largo Paolo Braccini 2, 10095, Grugliasco, Italy; b Department of Infectious Diseases, Istituto Superiore di Sanità, Viale Regina Elena 299, 00161, Rome, Italy; c Institute of Food Safety, Animal Health and Environment BIOR, Lejupes Street 3, Riga, LV-1076, Latvia; d University of Latvia, Jelgavas Street 1, Riga, LV-1004, Latvia

**Keywords:** Trichinella britovi, Freezing, Snow, Temperature, Relative humidity, Subnivium

## Abstract

Parasite nematodes of the genus *Trichinella* are transmitted from one host to another through the ingestion of larvae present in striated muscles. The longer the survival of muscle larvae in host carcasses, the higher the probability of being ingested by a scavenging host. Thereby, these nematodes developed an anaerobic metabolism favouring their survival in decaying tissues. In addition, muscle larvae of three taxa, namely *Trichinella nativa*, *Trichinella britovi* and *Trichinella* T6, can survive freezing for several months to several years depending on the taxon. The aim of the present work was to investigate the survival time of *T. britovi* larvae in naturally infected host carcasses preserved beneath or above the snow. Fox and raccoon dog carcasses naturally infected with *T. britovi* larvae were preserved beneath or above the snow in a cold mountainous area. Temperature and relative humidity were recorded. Every 14 days, muscle samples collected from each carcass, were digested and larvae were counted and given per os to laboratory mice to evaluate their reproductive capacity index (RCI). The RCI of larvae in carcasses preserved beneath the snow (the subnivium) ranged from 23 to 25 at day 0, to 12–18 after 112 days. In contrast, the RCI of larvae in carcasses preserved above the snow ranged from 22 to 27 at day 0, to 0.0 after 112 days. The difference between the RCIs of larvae beneath the snow and above the snow was statistically significant (*P* < 0.01). These data corroborate the hypothesis that the subnivium with its environmental stability favours the survival of *Trichinella* larvae in host muscles, increasing the probability of their transmission to other hosts. On the other hand, the environment above the snow, characterized by sudden temperature variations, causes strong environmental stress for larvae in host carrions causing their death.

## Highlights

• The snow cover increases the survival time of *Trichinella* larvae in host carrions.
• The subnivium, seasonal refuge of *Trichinella* larvae in host carcasses.
• Frequent thermal variations reduce larva survival in host muscles.
• Muscle putrefaction did not adversely affect the survival of *Trichinella* larvae.

## Graphical abstract

**Figure:**
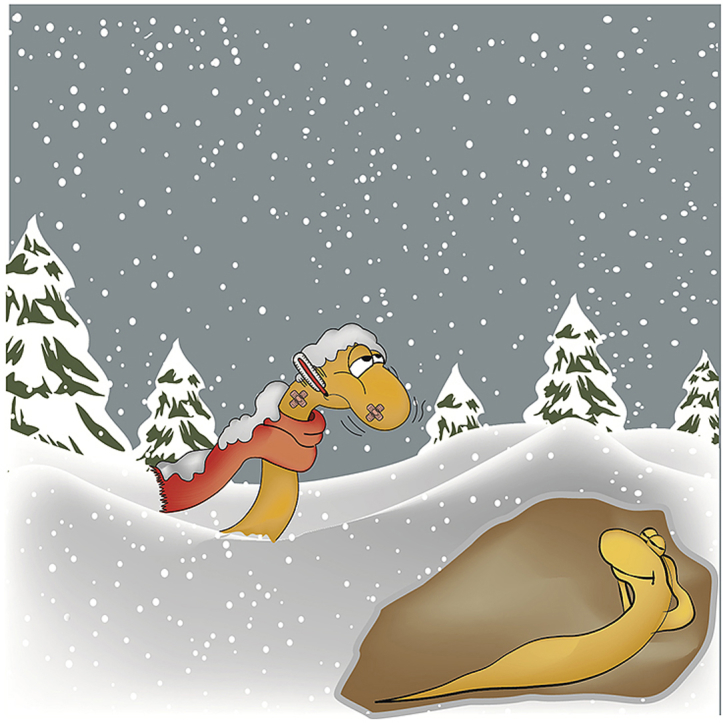

## 1 Introduction

Nematodes of the genus *Trichinella* are zoonotic parasites with a cosmopolitan distribution. The twelve taxa recognized so far in the genus are separated into two clades, one that encompasses species that encapsulate in host muscle tissues following muscle cell reprogramming, and a second that includes non-encapsulated species (Pozio and Zarlenga, 2013; Korhonen et al., 2016). Of the six encapsulated species, *Trichinella spiralis* which probably originated in Eastern Asia, shows a cosmopolitan distribution in tropical and temperate regions due to its passive introduction into Europe, North and South America and New Zealand (Pozio and Zarlenga, 2013). The geographical range of the other five encapsulated species shows a north-south cline. *Trichinella nativa* occurs in arctic and subarctic areas of the Holarctic region, approximately up to the isotherm – 4 °C in January in the south; *Trichinella britovi* is found in the Palearctic region from the isotherm −6 °C in January in the north up to North and Western Africa in the south; *Trichinella murrelli* is present in temperate areas of the Nearctic region; *Trichinella nelsoni* occurs in the Ethiopian region; and *Trichinella patagoniensis* in the Neotropic region (Pozio and Zarlenga, 2013).

These zoonotic nematodes are transmitted from one host to another through the ingestion of striated muscle tissues infected with larvae (Pozio and Murrell, 2006); however, vertical transmission has also been experimentally demonstrated in some rodent species and ferret, but not in foxes and pigs (Webster and Kapel, 2005). The most important reservoir hosts of *Trichinella* nematodes are those with a scavenger behaviour (Pozio and Murrell, 2006).

An important adaptation of *Trichinella* spp. muscle larvae, which facilitates parasite transmission, is a physiological mechanism to survive in decaying carcasses; the greater the persistence of larval viability, the higher the probability of being ingested by a scavenging host. Despite the larva-induced angiogenic process that develops around the nurse cell after larval penetration of the muscle cell, larval metabolism is basically anaerobic, which favours its survival in decaying tissues (Pozio, 2016).

The distribution areas of *T. nativa* and its related *Trichinella* T6 genotype, and *T. britovi* overlap, completely (*T. nativa* and *Trichinella* T6) or partially (*T. britovi*), with cold regions and the muscle larval stage of these taxa have developed mechanisms to survive in frozen carrions for several months (*T. britovi*) up to several years (*T. nativa* and *Trichinella* T6) (Pozio, 2016).

Previous studies on freezing temperature favouring the survival of *T. britovi* and *T. nativa* larvae in muscle tissues of naturally or experimentally infected host carrions had shown that the optimal freezing temperature range for survival corresponds to temperatures between 0 °C and −20 °C (Malakauskas and Kapel, 2003; Pozio, 2016). Furthermore, the molecular identification of *Trichinella* spp. larvae showed that when infected muscle tissues are frozen and thawed more than one time, a DNA degradation occurs, which is caused by thermal shock (Pozio and La Rosa, 2010). Based on these data, Pozio (2016) hypothesized that the habitat under the snow, i.e. the subnivium, could represent the ideal haven for the survival of *Trichinella* larvae in decaying muscles of host carcasses, since it provides environmental stability.

The aim of the present study was to investigate the survival and infectivity of *T. britovi* larvae in muscle tissues of naturally infected carnivore carcasses preserved beneath and above the snow.

## 2 Materials and methods

### 2.1 Biological samples

Two carcasses of foxes (*Vulpes vulpes*) and a carcass of a raccoon dog (*Nyctereutes procyonoides*) were collected within the Latvian State programme for the Control and Eradication of Rabies (Monitoring Plan of Animal Infectious Diseases, 2017). Animals were first tested for rabies and only rabies negative animals were subsequently used to test for *Trichinella* sp. infection at the Institute of Food Safety, Animal Health and Environment BIOR, Riga, Latvia. Animals were hunted between the end of November and the beginning of December 2017. Carcasses were eviscerated and 25 g of muscles from the tongue and diaphragm pillars were collected and submitted to artificial digestion according to the protocol of the Commission Regulation 1375/2015 (European Commission, 2015). Following digestion, larvae were washed and counted in triplicate to detect the number of larvae per g (LPG) (Table 1). Then each carcass was packaged in plastic bags, placed in a polystyrene box containing ice packs and forwarded to the Department of Veterinary Sciences, University of Turin, Grugliasco, Italy, by an international courier on December 11, 2018. The carcasses were delivered on December 12, 2017. Upon arrival, the temperature in the polystyrene boxes was ascertained to be 6 °C. The raccoon dog carcass was cut in two symmetrical parts with a longitudinal cut along the spine, and henceforth considered as two carcasses. Carcasses were stored at +4 °C and transported on December 13, 2017 in polystyrene boxes containing ice packs to the locality where they were to become the object of the experimental study.

**Table 1:** Features of the animals infected by *Trichinella britovi* used in this study.

Carnivorous species	Age	Hunting data	District of origin in Latvia	LPG^a^
Raccoon dog	4 yrs	29.11.2017	Apes	58.2
Red fox	1 yr	02.12.2017	Madonas	15.6
Red fox	1 yr	05.12.2017	Alūksnes	18.1

a LPG, average number of larvae per gram from the tongue and diaphragm pillars.

### 2.2 Geographical and habitat features of the locality of carcass storage

The study was carried out in the Alps at 1175 m above sea level (asl). The scavenger proof box where the fox and raccoon dog carcasses were preserved, was placed in a locality of the Oulx municipality (longitude 45°02′40″N; latitude 6°47′30″E), Turin province, Northern Italy. The box was positioned in a restricted open space exposed to the north and shadowed by a building on the south side. The surrounding area constituted the south slope of a large inner alpine valley (Susa Valley) mainly covered by a Scots pine (*Pinus sylvestris*) forest interspersed with juniper (*Juniperus communis*), ash (*Fraxinus excelsior*) and birch (*Betula pendula*).

### 2.3 Carcass preservation beneath and above the snow

**Fig. 1:**
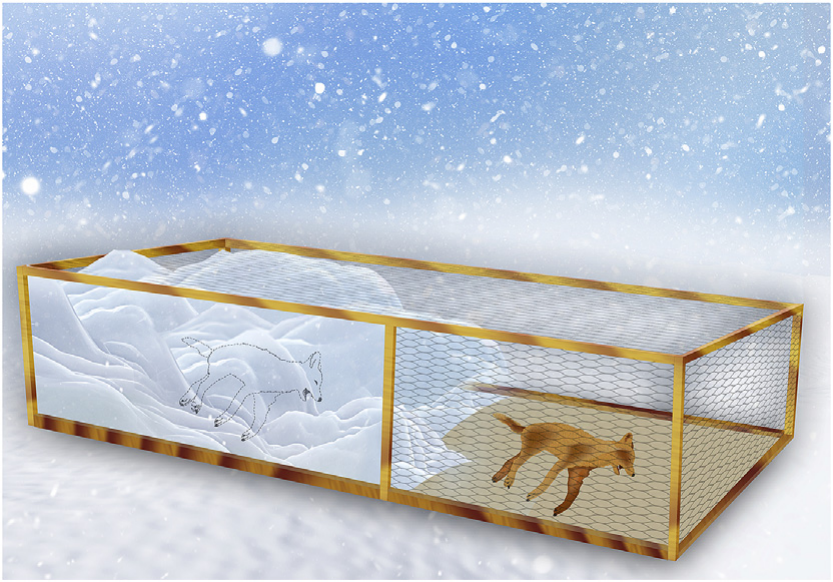
Box containing the animal carcasses beneath and above the snow.

A wooden frame measuring 90 × 80 × 50 cm with a 2 × 2cm netted mesh was used to house the carcasses. It was placed on the ground and surrounded by snow (Fig. 1). A fox carcass and a racoon dog carcass were placed on one side of the bottom of the box. Then, half of the box was filled with snow almost to the upper border and, if necessary, snow was added to maintain the depth at about 45–50 cm. The second fox carcass and the second raccoon dog carcass were placed on the bottom of the other half of the box without any snow cover (Fig. 1). A wooden partition prevented the snow from falling into the second half of the box.

### 2.4 Recording of temperature and humidity

A temperature and humidity recording systems were used to register the temperature and humidity of the carcasses beneath and above the snow. The probes were placed in contact with skin and a data logger was set up to register the maximum and minimum temperatures and humidity every 12 h.

### 2.5 Muscle sample collection

Muscle samples (25 g) were collected nine times from the same muscles of each carcass every 14 days from December 18, 2017 (day 0), up to April 9, 2018 (day 112). Muscle samples were preserved in a 50 ml Falcon tube and forwarded in a polystyrene box containing ice packs to the European Union Reference Laboratory for Parasites (EURLP) (Istituto Superiore di Sanità, Rome, Italy), for molecular identification of *Trichinella* species and the detection of the reproductive capacity index (number of larvae collected from the mouse carcass/number of larvae given per os; RCI) of *Trichinella* larvae in mice.

### 2.6 Detection of *Trichinella* larva infectivity and species

Muscle samples of each carcass were digested according to the Regulation (EU) 1375/2015 (European Commission, 2015). Following digestion, larvae were washed in PBS and counted in triplicate. Ten larvae were preserved in alcohol for species identification by multiplex PCR according to a published protocol (Pozio and La Rosa, 2010). Then, 200 larvae/mouse were injected per os to 1–3 Swiss CD1 female mice of 20 g. Mice were euthanized 40 days post infection (p.i.) and skinned and eviscerated carcasses were digested individually. After digestion, larvae were washed in PBS and counted in triplicate. Animals were housed and treated according to the European Directive on laboratory animal welfare (European Commission, 2010 and L.D. 26/2014) and the protocol was approved by the Italian Ministry of Health (DL 116/92).

### 2.7 Statistical analysis

Mean RCI values between the two groups (carcasses beneath and above the snow) on different days, were evaluated by a linear regression model including days spent within the carcasses beneath/above snow and their interactions by StataCorp. (2013). Stata Statistical Software: Release 13. College Station, TX: StataCorp LP. This allowed the evaluation of *P*-values for each sampling day comparing the beneath/above snow groups. *P* < 0.01 was regarded as statistically significant.

## 3 Results

All larvae (n = 360) collected from the four carcasses for species identification were identified as *T. britovi*. These larvae represented approximately 1.3% of the total number (n = 27,747) of larvae collected from the four carcasses during the study period.

**Fig. 2:**
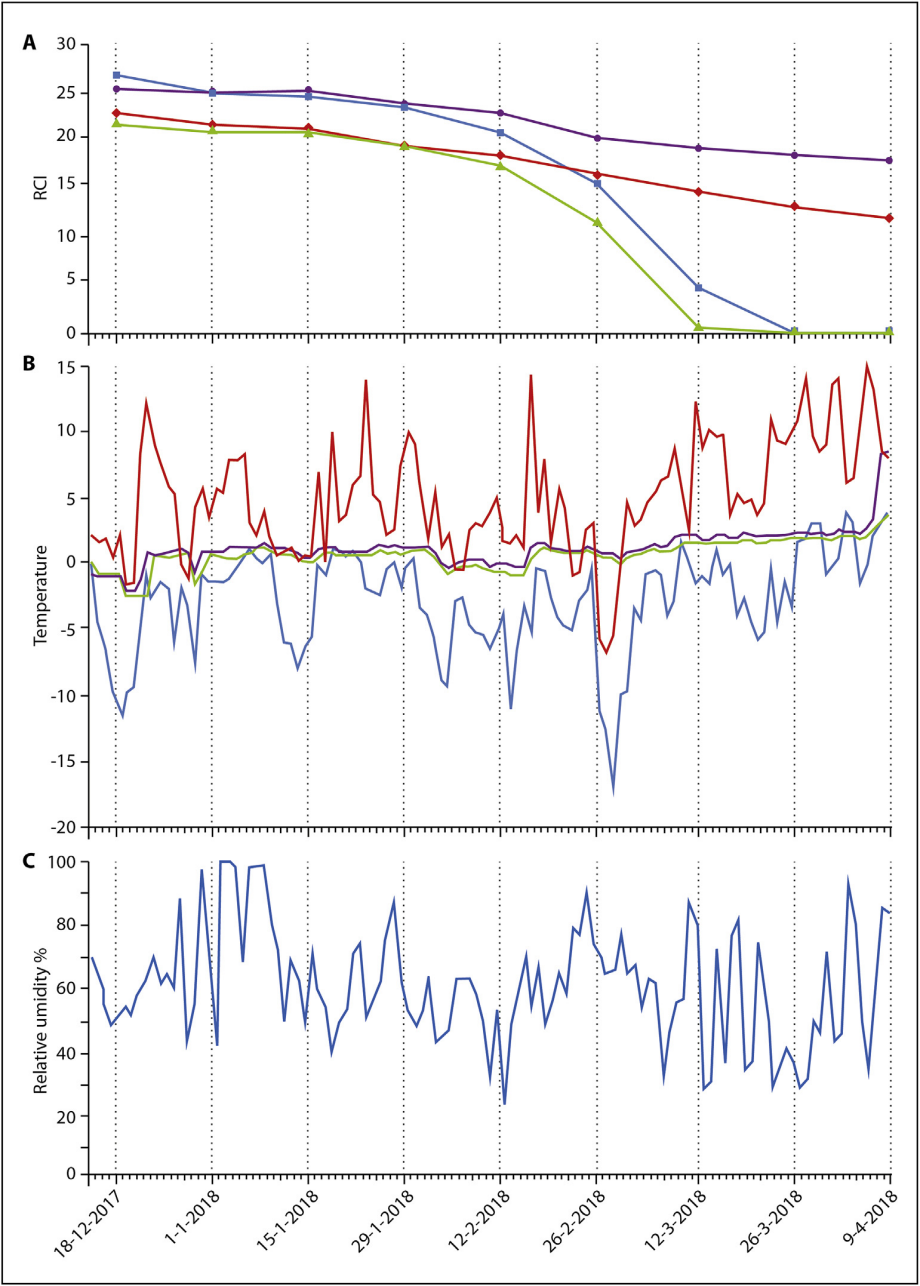
Reproductive Capacity index (RCI) of *Trichinella britovi* larvae, minimum and maximum temperature and relative humidity of fox and raccoon dog carcasses stored beneath and above the snow during the study period. Panel A, RCI of *T. britovi* larvae present in muscles of fox carcasses stored below (red line) or above (green line) the snow and of the raccoon dog carcasses stored below (violet line) or above (light blue line) the snow. Panel B, minimum (green line) and maximum (violet line) temperature below the snow and minimum (light blue line) and maximum (red line) daily temperature above the snow. Panel C, daily relative humidity (%) of carcasses above the snow. (For interpretation of the references to colour in this figure legend, the reader is referred to the Web version of this article.)

An average of 372 larvae were collected from the fox carcass stored beneath the snow every 14 days and their RCI in mice was reduced by 47.4% during the study period. An average of 1232 larvae were collected from the raccoon dog carcass stored beneath the snow every 14 days and their RCI in mice was reduced by 30.1% during the study period (Table 2; Fig. 2A). An average of 211 and 1283 larvae were collected from the fox and raccoon dog carcasses stored above the snow every 14 days, respectively, and their RCIs in mice were reduced by 100% during the study period (Table 2; Fig. 2A). Up to the 56th day of the study period, no statistical differences were observed between the mean RCI values of larvae collected from the carcasses beneath and above the snow. From the 70^th^ to the 112^th^ day of the study period, the mean RCI values of larvae collected from carcasses beneath and above the snow were statistically significant (*P* < 0.01) (Table 2).

**Table 2:** Reproductive capacity index (RCI) of *Trichinella britovi* larvae of animal carcasses stored above and below the snow.

Date	N. of days	RCI (N. mice)				*P*^a^
		Under the snow		Above the snow		
		Fox carcass	Raccoon dog carcass	Fox carcass	Raccoon dog carcass	
December 18	0	23 (1)	25.6 (3)	22 (1)	26.8 (3)	0.65
January						
01	14	21.5 (2)	24.9 (3)	21 (1)	25.1 (3)	0.71
15	28	21 (1)	25.1 (3)	20.9 (1)	24.6 (3)	0.66
29	42	19.3 (2)	23.7 (3)	20.1 (1)	23.3 (3)	0.95
February						
12	56	18.4 (1)	22.8 (3)	17.3 (1)	20.9 (3)	0.41
26	70	16.6 (1)	20.2 (3)	11.1 (1)	15.4 (3)	<0.01
March						
12	84	15.1 (1)	19.6 (3)	0.6 (1)	4.7 (3)	<0.01
26	98	13.2 (1)	18.7 (3)	0.00 (1)	0.2 (3)	<0.01
April						
09	112	12.1 (1)	17.9 (3)	0.00 (1)	0.00 (3)	<0.01

a Between mean RCI values of the two groups of carcasses below the snow and above the snow.

During the study period, the average minimum and maximum temperature in the carcasses beneath the snow were from −0.6 °C to +1.9 °C (range −2.5/+2.4) and from 0.0 °C to +2.4 °C (range −2.1/+3.3), respectively (Table 3; Fig. 2B). The average minimum and maximum temperature in the carcasses above the snow were from −5.6 °C to +1.5 °C (range −16.9/+3.9) and from +1.9 °C to +10.4 °C (range −7.1/+14.8), respectively (Table 3; Fig. 2B). It follows that the temperature variation in the subnivium was 5.5 times lower than that above the snow. During the study period, the average relative humidity in the carcasses above the snow ranged from 53.0% to 68.1% (range 23.9%–99.9%) (Fig. 2C), whereas, the average relative humidity in the carcasses beneath the snow remained stable at 97.9%–100%.

**Table 3:** Deviation between the maximum and the minimum daily temperature (°C) in the carcasses below and above the snow in the study period of 112 days.

Month	Below the snow		Above the snow	
	Average minimum temperature (range)	Average maximum temperature (range)	Average minimum temperature (range)	Average maximum temperature (range)
December^a^	−0.6 (−2.5/+0.6)	0.0 (−2.1/+1.0)	−4.7 (−11.7/-1.0)	+4.2 (−1.7/+8.9)
January	+0.6 (0.0/+1.1)	+1.0 (+0.2/+1.4)	−1.6 (−7.9/+0.9)	+4.7 (+0.2/+13.8)
February	+0.1 (−0.9/+1.1)	+0.5 (−0.4/+1.4)	−5.6 (−16.9/-0.5)	+1.9 (−7.1/+14.2)
March	+1.4 (−0.1/+1.9)	+1.8 (+0.4/+2.3)	−2.0 (−10.0/+3.1)	+6.0 (−0.1/+13.8)
April^b^	+1.9 (+1.7/+2.4)	+2.4 (+2.1/+3.3)	+1.5 (−1.8/+3.9)	+10.4 (+6.0/+14.8)

a From 19 to 31 December.

b From 1 to 7 April, excluding the last two days of the study period (8 and 9 April), when the snow was completely melted.

## 4 Discussion

This study was conducted for 112 days during which the RCI values of *T. britovi* larvae in fox and raccoon dog carcasses were strongly influenced by the environmental parameters (temperature and relative humidity) of the habitat both beneath and above the snow. Initially, the RCI values of larvae collected from the four carcasses ranged from 26.8 to 22.0. At the end of the study, the RCI values of larvae collected from the two carcasses preserved in the subnivium showed a percentage of reduction of 30.1% and 47.4%, whereas the RCI values of larvae collected from the two carcasses preserved above the snow and consequently subjected to strong and sudden variations in temperature and relative humidity, showed a percentage of reduction of 100%, i.e. larvae did not infect mice.

The environmental stability of the subnivium favours the survival of *Trichinella* larvae in host muscles, increasing the probability for their transmission to other hosts. The temperature range of the subnivium is influenced by the snow cover height and snow density. Nevertheless, the temperature of the subnivium does not fall below −10 to −20 °C even if the ambient temperature drops below −50 °C (Pauli et al., 2013). This temperature range could explain why *T. nativa* larvae and, to a lesser degree, *T. britovi* larvae in host carcasses are able to survive up to −20 °C (Pozio, 2016). Lower temperatures and frequent thermal variations do not allow the survival of muscle larvae in the collagen capsule present in the host muscles. Similarly, the subnivium represents a seasonal refuge permitting a diversity of organisms to survive extreme winter temperatures (Bjork and Molau, 2007; Korslund and Steen, 2006; O'Connor and Rittenhouse, 2016; Whitaker and Stauffer, 2003; Hagvar, 2010). The formation of the subnivium results from continual sublimation and condensation occurring within a snowpack and the upward migration of water vapour from areas of high vapour density (closest to the ground) to low vapour density (near the snow surface) (Marchand, 2013; Pinzer and Schneebeli, 2009). This movement of vapour reduces the size of the ice crystals in the lowermost snow layer, creating a network of loosely-connected crystals whose low-density traps heat released from the soil. When snow depths are sufficiently high, the low thermal conductivity of the snowpack insulates the subnivium, creating a warmer and more stable microclimate compared to external air temperatures (Pruitt, 2005). In our experimental study, the temperature variation of carcasses in the subnivium ranged from −2.5 °C to +3.3 °C with a maximum delta of 5.8 °C and a relative humidity close to 100%. In contrast, the temperature variation of carcasses exposed above the snow was in the range of −16.9 °C to +14.8 °C with a maximum delta of 31.7 °C and a relative humidity ranging from 23.9% to 99.9%.

In the present study, the temperature variation in the subnivium was 5.5 times lower than that above the snow. Snow accumulation and density, both of which directly control the formation and persistence of the subnivium, are affected by ambient temperature, wind, snowfall, and radiation fluxes (Harestad and Bunnell, 1981). Deep, low density snow is most effective in maintaining the thermal stability of the subnivium (Ge and Gong, 2010). With warmer ambient temperatures, ablation increases, reducing depth and increasing snow density throughout the entire snowpack. Under colder conditions, the temperature gradient between the bottom layer of snow and the air temperature increases, which increases snow density at the surface of the snowpack (Kim and Jamieson, 2014). Despite increased density at the surface, air temperatures at or below 0 °C prevent the occurrence of surface snow melt and support the retention.

**Fig. 3:**
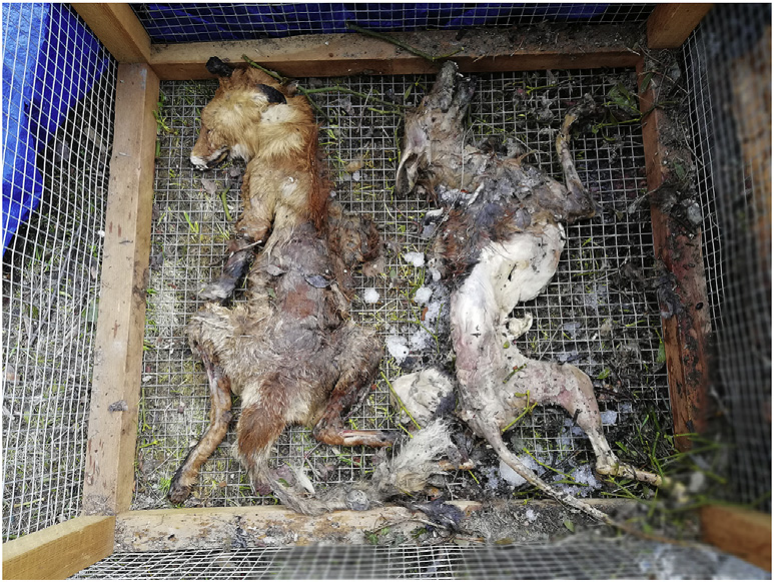
Fox carcasses preserved above the snow (on the left) and beneath the snow (on the right) after 112 days.

At the end of the study, carcasses preserved in the subnivium appeared more degraded than carcasses stored above the snow (Fig. 3). However, the putrefaction of muscles did not adversely affect the survival and infectivity of *Trichinella* larvae as previously observed (Pozio, 2016).

This study was conducted at 1175 m asl on the southern slope of the Alps, an area where *Trichinella* infection was widespread in wild carnivores (Marazza et al., 1960). However, during the last decades, a reduction of *T. britovi* prevalence was observed in the entire Alps region (Di Blasio et al., 2018; Pozio E. and Rossi L., unpublished data). At the same time, the snow depth and snow cover in the Alps showed a significant decrease for elevations below 1300 m asl (Marty, 2013; Scherrer et al., 2004). In Latvia, a relationship between *T. britovi* infection in wild boar and snow cover was also documented (Kirjušina et al., 2015).

Even if latitude, land cover, and interannual variability, influence the subnivium phenology, optimal subnivium conditions depend on the relationship between air temperature, snow depth, and snow density. Above-freezing temperatures promote rapid subnivium establishment, while below-freezing temperatures reduce snowmelt and support longer subnivium maintenance periods. In the Alps, the number of days with snow cover of at least 30 cm reduced from 60 in the eighties to less than 30 from the nineties of the last century (Marty, 2013). Colder air temperatures are required to promote subnivium maintenance. Warmer air temperatures increase ablation, which reduces depth, increases density, and reduces the overall insulative capacity of snow cover (Ge and Gong, 2010). Future climate change scenarios predict warmer winter temperatures, which are often accompanied by an increase in precipitation falling as rain rather than snow and an overall increase in air temperature variability. Disturbances to the subnivium in either extent, duration, or thermal stability can therefore disrupt the regimes that currently provide fitness benefits to a variety of organisms, including cold adapted representatives of the genus *Trichinella*, resulting in phenological mismatches and enhanced mortality (Schimel et al., 2004).

In conclusion, the subnivium with its environmental stability represents a seasonal refuge for *Trichinella* larvae in host carrions and even if carcasses preserved in the subnivium appeared more degraded than carcasses stored above the snow, the putrefaction of muscles did not adversely affect the survival and infectivity of the infecting stage of this parasite. The present study demonstrates the interaction between environmental conditions and the life cycle of *Trichinella* nematodes, which apparently do not show a free-living stage.

## Declarations of interest

None.
